# Use of Natural Diversity and Biotechnology to Increase the Quality and Nutritional Content of Tomato and Grape

**DOI:** 10.3389/fpls.2017.00652

**Published:** 2017-05-12

**Authors:** Quentin Gascuel, Gianfranco Diretto, Antonio J. Monforte, Ana M. Fortes, Antonio Granell

**Affiliations:** ^1^Laboratory of Plant-Microbe Interactions, Centre National de la Recherche Scientifique, Institut National de la Recherche Agronomique, Toulouse UniversityCastanet Tolosan, France; ^2^Italian National Agency for New Technologies, Energy, and Sustainable Development, Casaccia Research CentreRome, Italy; ^3^Instituto de Biología Molecular y Celular de Plantas, Agencia Estatal Consejo Superior de Investigaciones Científicas, Universidad Politécnica de ValenciaValencia, Spain; ^4^Faculdade de Ciências de Lisboa, Instituto de Biossistemas e Ciências Integrativas (BioISI), Universidade de LisboaLisboa, Portugal

**Keywords:** fruit quality, germplasm, grape, omics, new plant breeding techniques, tomato, QTLs

## Abstract

Improving fruit quality has become a major goal in plant breeding. Direct approaches to tackling fruit quality traits specifically linked to consumer preferences and environmental friendliness, such as improved flavor, nutraceutical compounds, and sustainability, have slowly been added to a breeder priority list that already includes traits like productivity, efficiency, and, especially, pest and disease control. Breeders already use molecular genetic tools to improve fruit quality although most advances have been made in producer and industrial quality standards. Furthermore, progress has largely been limited to simple agronomic traits easy-to-observe, whereas the vast majority of quality attributes, specifically those relating to flavor and nutrition, are complex and have mostly been neglected. Fortunately, wild germplasm, which is used for resistance against/tolerance of environmental stresses (including pathogens), is still available and harbors significant genetic variation for taste and health-promoting traits. Similarly, heirloom/traditional varieties could be used to identify which genes contribute to flavor and health quality and, at the same time, serve as a good source of the best alleles for organoleptic quality improvement. Grape (*Vitis vinifera* L.) and tomato (*Solanum lycopersicum* L.) produce fleshy, berry-type fruits, among the most consumed in the world. Both have undergone important domestication and selection processes, that have dramatically reduced their genetic variability, and strongly standardized fruit traits. Moreover, more and more consumers are asking for sustainable production, incompatible with the wide range of chemical inputs. In the present paper, we review the genetic resources available to tomato/grape breeders, and the recent technological progresses that facilitate the identification of genes/alleles of interest within the natural or generated variability gene pool. These technologies include omics, high-throughput phenotyping/phenomics, and biotech approaches. Our review also covers a range of technologies used to transfer to tomato and grape those alleles considered of interest for fruit quality. These include traditional breeding, TILLING (Targeting Induced Local Lesions in Genomes), genetic engineering, or NPBT (New Plant Breeding Technologies). Altogether, the combined exploitation of genetic variability and innovative biotechnological tools may facilitate breeders to improve fruit quality tacking more into account the consumer standards and the needs to move forward into more sustainable farming practices.

## Introduction

Since the dawn of agriculture in Neolithic communities some 12,000–10,000 years ago, the selection of plants exhibiting the most desirable traits has never ceased. This, so-called, domestication process appears to have been instrumental in our ancestors' transition from a hunter-gatherer to an agricultural lifestyle (Gepts, 2014), and was characterized by the low number of plant species to succeed as widely-grown crops in modern societies. Initially an intuitive process, selection was made on a few easy-to-observe desirable traits (e.g., fruit size, shape and color, or seed quality; Chalhoub et al., 2014; Vogel, 2014). As in species reduction, only a few genes exercising large phenotypic effects within this limited number of species were selected (Tang et al., 2010).

In fruit crops, initial selection was probably based on nutritious, non-toxic, and palatable features. Hedonic and culinary qualities, including flavor, succulence, juiciness, and other consumer-desirable characteristics were added later (Table 1). However, since the 1930s breeders, including tomato breeders, have centered their efforts on productivity and have basically neglected fruit quality, including traits of interest to consumers (e.g., flavor or nutritious). This can be explained in many ways: one is the fact that it is difficult to breed for complex multigene traits such as flavor; another is our lack of understanding of the molecular genetic basis of fruit quality (Klee, 2010; Lim et al., 2014). Together with changes in consumer habits, this has led to lower fruit quality and loss of flavor, which indirectly have a negative impact on fruit consumption (Klee, 2010; Orzaez et al., 2010). Hence, scientists and breeders are faced with a real challenge to improve grapes and tomatoes so that they meet the needs both of producers, i.e., productivity, and consumers, i.e., taste and healthiness (Handa et al., 2014). The relevance of this goal lies in the importance of nutrition (i.e., vitamins, antioxidants, and minerals) to remedy physiological disorders and reduce the incidence of human diseases (Klee, 2010). Today, regarding what quality parameters are crucial to improve, yield, and sustainability are the first, because of their role to ensure food security and healthiness. So, we need to maintain the yield per hectare, reducing fertilizers, and pesticides and increasing resilience to biotic and abiotic stresses in a global climate change scenario. The next objective should be increasing nutritional content, especially for crops that will be cultivated in poor areas. Enable crop diversification in poor areas could be a solution. Moreover, depending on the crop, different nutritional contents will be easier to increase. In the case of tomato, carotenoid related compounds are a clear target. For grapes, polyphenols are the main topic of studies. Finally, consumer preferences and taste should be taking into account.

**Table 1:** **Quality standards according to the different stakeholders in the Agri-Food chain**.

**Standards**	**Quality traits**
Producer	Resistant against biotic and abiotic stresses.
	High yield (size…).
	Easy to harvest and handle.
	Synchronization of flowering or flowering time.
Market	Shelf-life.
	Less prone to handling and shipping damages.
	Biochemical products (soluble solid concentration for processing tomatoes, resveratrol for grapes).
Consumer	Flavor/succulent/juicy.
	Crispness/chewiness/oiliness.
	Appearance/color.
	Healthy/sustainably produce.
	Nutritious.
Environmental	Reduction of synthetic fertilizers and pesticides.

Grape (*Vitis vinifera* L.) and tomato (*Solanum lycopersicum* L.) are the focus of the present review. Both produce fleshy, berry-type fruit, and have undergone important domestication and selection processes that have dramatically reduced their genetic variability. Tomato and grapevine have been selected to satisfy the quality standards required by humans. This has entailed a preference for varieties that were more productive, gave larger fruits or displayed defined organoleptic characteristics. In grapevine, despite the thousands of cultivars available, the market is dominated by a few and these are classified as a function of the final product: table grapes or raisins, or their use in winemaking (This et al., 2006). In tomato, there has also been a progressive/dramatic reduction in variability during the domestication process in the original centers of diversification and, later, when introduced into Europe, and then reintroduced into North America (Blanca et al., 2015). Initially, selection was performed by farmers; later, breeders and researchers became involved. Ultimately, this has led to the development of tomato cultivars yielding fruits of the shape, color, and size of choice. For a long time, tomatoes have been used both as a fresh product and as a processed commodity in soups, juices, sauce, pastes, powders, or concentrates, all of which require different characteristics (Bai and Lindhout, 2007; Bergougnoux, 2014). While grape and tomato share a past history of reduced variability, important differences exist: loss of flavor has more dramatically affected tomato, in part, due to more active breeding for productivity than in grapevine. Knowledge of the molecular genetic basis of fruit quality traits and of environmental impact on these traits will facilitate the maintenance of and/or an increase in production while enabling us to improve or change flavor at will.

Despite the biotechnological advances of recent decades, breeding programs often fail when dealing with complex quality traits (Handa et al., 2014). Progress in biotechnology and omics technologies applied to the variability available are likely to help us decode the underlying genetic basis of complex traits. Best alleles could subsequently be transferred into cultivars by crossing, genetic engineering, or NPBT (New Plant Breeding Technologies), to improve the quality of tomato or grape fruit. The present review is based on four fundamental approaches to increase fruit quality: (i) to enhance/maintain germplasm diversity as the source for best alleles; (ii) to understand the biochemical and genetic basis of fruit quality traits using this genomic and phenotypic diversity; (iii) to develop and use tools to dissect fruit quality traits, including improved computational technologies and network analysis; and (iv) to conduct functional studies of cultivar improvement. In conclusion, we will present an up-to-date view of the genetic resources and technologies that can improve fruit quality.

## The contributions of biotechnological tools to link genomic variability present in *in-situ* and *ex-situ* germplasm collections with the derived phenotypic diversity

### Germplasm diversity

Sources of germplasm, here defined as the collection of genes and their alleles available for plant improvement, include cultivated species and sexually-compatible wild species but could also include sexually-incompatible species harboring genes that can impact on fruit quality and be transferred through genetic engineering. Only a minimal part of the wide variability present in wild germplasm was domesticated and resulted in selective gain of phenotypical or physiological traits of interest for humans. Similarly, the domestication process also resulted in a loss of genes that were left behind in non-selected wild relatives, but were needed to improve crop adaptation to environmental changes. Modern plant breeding programs are based on a process of human selection which differs dramatically from that of natural evolution: selective pressure is no longer defined primarily by a multifactorial changing environment but by narrow human standards that focus on a few traits. Hence, even if the number and nature of genes under selection may vary across the different domesticated species, phenotypic, and genetic diversity are more heavily reduced in “domesticated” germplasm than in their wild relatives. These so-called bottlenecks occurred during domestication and cultivar development, and have recently been confirmed by sequencing (Tang et al., 2010; Abbo et al., 2014; Amini et al., 2014; Andersen et al., 2015). This reduction in genetic variability is particularly evident in cultivated grapevine, in part, as a consequence of its vegetative propagation (Roby et al., 2014), but it also occurs in tomato. On the whole, as the (agronomical) traits selected by humans differed from those oriented toward optimal adaptation to the natural environment, a clear dichotomy arose between crops and their wild progenitors (Gepts, 2014). This particular genetic bottleneck, known as genetic erosion, could compromise modern cultivars as they may be unable to cope with global warming or newly emerging diseases (Prada, 2009; Chen et al., 2013; Bai et al., 2016). For instance, the wild North American grapevine species *Muscadinia rotundifolia* is known to be resistant to both powdery and downy mildew (Feechan et al., 2013). This resistance was mapped to a single locus that contains a family of seven *TIR-NB-LRR* genes known to be involved in effector-triggered immunity. Therefore, these wild species could constitute a source of resistance-related genes to be introgressed into susceptible cultivars.

In light of the consequences of genetic erosion and the importance of preserving sources of genetic and phenotypic diversity in crops, the scientific community has developed germplasm banks (Prada, 2009). Nowadays, there are more than a thousand seed banks distributed all over the world. Tomato genetic resources in gene banks have been reviewed by (Bai and Lindhout, 2007; Di Matteo et al., 2011) (Table 2) and altogether may account for over 20,000 accessions. Grapevine germplasm also exhibits great diversity with up to 10,000 cultivars predicted (Laucou et al., 2011). In this context, many seed centers have been dedicated specifically to grapevine species—especially in countries with a tradition of viticulture (Table 2). Furthermore, the Svalbard Global Seed Vault conserves in permafrost the seeds of over four thousand plant species (>774,601 accessions, of which 7,382 correspond to tomato or wild relatives of tomato clade) (www.seedvault.no) (Fowler, 2008; Westengen et al., 2013).

**Table 2:** **Main seed bank collections worldwide where tomato and grapevine germplasm can be found**.

**Name**	**Plants**	**Resources**	**Website**	**References**
The Solanaceae database	Non-tuberous Solanaceae germplasm collection	*Ex situ* plant collections	http://www.bgard.science.ru.nl/	Bai and Lindhout, 2007
The isogenic tomato ‘mutation library’	Solanaceae	About 3,500 tomato monogenic mutants from the genetic background of the inbred variety M82 by treatment with EMS (ethyl methane sulfonate) and fast-neutron mutagenesis	http://zamir.sgn.cornell.edu/mutants/	Menda et al., 2004
French Network of Vine Conservatories	Grapevine (*Vitis vinifera* L.)	7,000 accessions from 40 countries	http://www1.montpellier.inra.fr/vassal/	French Network of Vine Conservatories
The EuropeanVitisdatabase	Grapevine (*Vitis vinifera* L.)	27,000 Vitis accessions from 13 european wine-growing countries	http://www.eu-vitis.de/index.php	
FAO/IAEA Mutant Variety Database (MVD)	Wide range of plant mutant including tomato and grapevine		http://mvd.iaea.org/#!Home	FAO/IAEA

Genetic resources include wild, landraces (heirlooms and old cultivars of local importance), modern cultivars, and synthetic populations, and constitute the ground material for breeders. Populations of wild relatives offer breeders untapped genetic and phenotypic diversity that has evolved over millions of years to adapt to a wide range of environmental niches (Honnay et al., 2012). It is very much in our interest to study this in depth (Khan et al., 2012). Landraces/heirlooms or traditional varieties represent old cultivars that may be of more or less local importance and were developed/selected by traditional farmers over hundreds or a few thousand years to best fit their needs. Landraces (local varieties) generally display greater diversity than modern cultivars as they have been selected to adapt to local, sometimes hostile environments, at a time when agronomic technology (i.e., irrigation, fertilizers, pesticides) was not yet widely available. Cultivar uniformity was not desirable when varieties had to successfully adapt to a range of environmental conditions (Fernie et al., 2006; Cebolla-Cornejo et al., 2013). Modern agronomic practices often result in more homogeneous environmental conditions: tomato cultivation in greenhouses entails controlled watering, facilitating the selection/development of genetically uniform cultivars to enhance yield performance. Hence, landraces constitute a source of allelic variants lost to modern breeding (i.e., over the last 80 years) but potentially available for variety improvement (Mazzucato et al., 2008; Prada, 2009; Leida et al., 2015). Because of their greater proximity to modern cultivars than their wild relatives, landrace cultivars with the desired phenotypes hold great potential for cultivar improvement (Zhu et al., 2008; Prada, 2009; Biasi and Brunori, 2015). For example, Corrado et al. (2013), studied variability in a set of 214 tomato accessions which included wild relatives, cultivated landraces, and commercial varieties. They identified a number of loci which were under strong positive selection among landrace and commercial cultivars. Although the diversity present in wild and landrace populations makes them useful for the identification of genotypes carrying genes of agronomic importance, they are of less use when we attempt to accurately dissect the underlying genetic basis. To overcome these difficulties, researchers and breeders have developed a wide range of cross populations such as Recombinant Inbred Lines (RILs), Near Isogenic Lines (NILs) or Introgression Lines (ILs), Double Haploid Lines (DHLs), Induced Mutant Lines (IMLs), TILLING (Targeting Induced Local Lesions in Genomes) Lines (TLs) (Varshney et al., 2014; Henikoff et al., 2004), Multiparent Advanced Generation Intercross (MAGIC, Cavanagh et al., 2008) and Nested Association Mapping (NAM, McMullen et al., 2009; Table 3). In grape, as in other perennial/long generation time and/or self-incompatible species, for which it is difficult or impossible to generate inbred lines, F_1_ segregating populations (also termed Cross-Pollinators, CP) have been developed for genetic mapping (Grattapaglia and Sederoff, 1994) and propagated by grafting. Finally, germplasm collection can also be used directly as a mapping population in Genome-Wide Association Studies (GWAS; Rosenberg et al., 2010).

**Table 3:** **Breeding populations developed in tomato and grape**.

**Name**	**Definition**	**Advantages/Disadvantages**	**Tomato and vine populations publicly available**	**References**
Recombinant inbred lines (RILs)	A Recombinant inbred line is developed by crossing inbred lines followed by repeated selfing up to create an inbred line whose genome is a mosaic of the parental genomes and total or nearly homozygous.	Due to the different events of recombination that happen in parental gametes, two RILs resulting from the same cross present different mosaics of the parental genomes. Hence, RILs populations allow to estimate the recombination rate between two genomic loci, constituting powerful tools for preliminary genetic mapping even for recessive traits. A relatively large number of generations are needed (>8), making difficult to be implemented in species with long generation time.	Several tomato RILs population were hosted by the Tomato Genetics Resource Center (TGRC), whereas grapevine population are quite rare, probably due very long generation time.	Broman, 2005; Carrera et al., 2012; Khan et al., 2012; Víquez-Zamora et al., 2014; Thapa et al., 2015
Near isogenic lines (NILs)/Introgression Lines (ILs)	NILs are a set of lines that are genetically identical, except for few loci, which result from several backcrosses between a donor line and an acceptor line, selecting at each generation the descendants with the trait of interest.	A population of Introgression Lines (ILs) is made of NILs in which introgression fragments cover the whole genome of the donor line. Introgression effects are evaluated in an elite genetic background, being ideal to introgress wild variability. The breeding scheme requires intensive marker assisted selection.	*S. penelli* and *S. habrochaites* IL collections are available at TGRC. *S. pimpinellifolium* ILs at CSIC.	Eshed and Zamir, 1995; Monforte and Tanksley, 2000; Barrantes et al., 2014
Multiparent Advanced Generation Intercross (MAGIC)	Recombinant Inbred Lines derived from the intercross of several genotypes (typically 8).	Multiple alleles are tested in a sible population, together with multiple recombinant events, thus providing a very high mapping resolution. The development of this populations is very time consuming, extensive genotyping is also needed and genetic analysis is complex.	Eight-way MAGIC population from four *S. lycopersicum* and four *S. lycopersicum* var. *cerasiforme* cultivars.	Pascual et al., 2015
Cross-Pollinator (CP) or F_1_ segregating population	Population consisted of full sibling plants after crossing two highly heterozygous genotypes.	The generation of this population is fast and inexpensive. Up to four alleles can be segregated. Only one meiosis has been carried out in each chromosome, so the mapping resolution is low.	Picovine × Ugni Blanc, Syrah × Pinot Noir.	Houel et al., 2015; Malacarne et al., 2015
Genome-wide Association studies	Collection of germplasm/cultivars that retain some extend of linkage disequilibrium.	Collections of germplasm are already available. The selection of the proper collection and the analysis is complex.	Major gemplasm banks are listed in Table 2.	Fodor et al., 2014; Ruggieri et al., 2014

**Figure 1:**
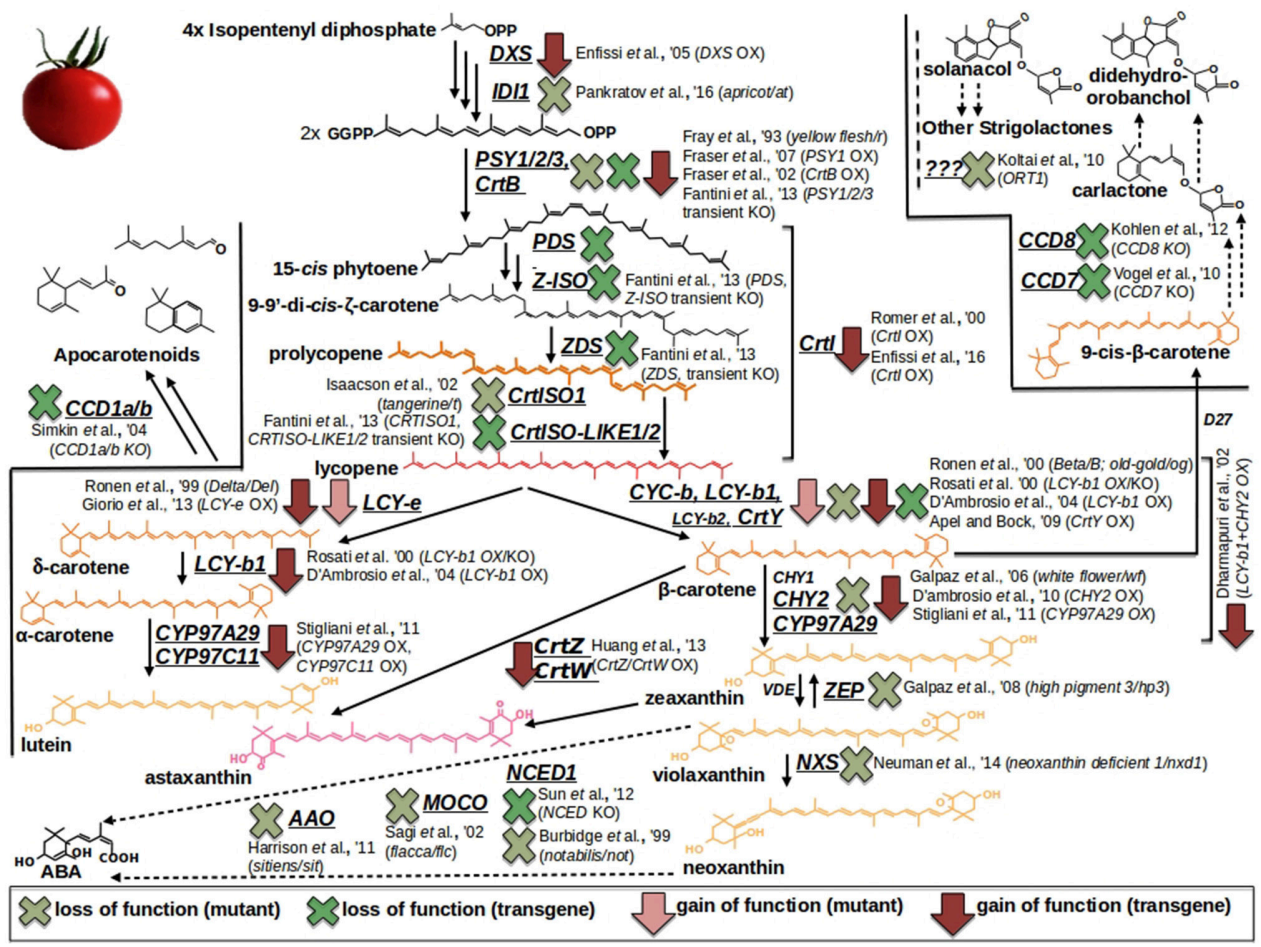
**Carotenoid/Apocarotenoid (Volatiles, VOCs; Abscisic acid, ABA; Strigolactones, SL) biosynthesis and overview of tomato natural (mutants) and metabolically engineered (ME) resources**. Light red arrows and light green crosses refer to, respectively, gain and loss of function mutants. Dark red arrows and dark green crosses pinpoint overexpression and knockout ME interventions, respectively.

One way to unravel the genetic basis of fruit quality traits is by analyzing spontaneous/natural or induced mutant lines (Di Matteo et al., 2011; Bauchet and Causse, 2012). For tomato, several natural mutants have been identified but these resources are limited in comparison with induced mutant collections (Bauchet and Causse, 2012; and Table 1). The carotenoid pathway, for example, is one of the best elucidated metabolism in tomato fruit due to the availability of a series of well-characterized mutations (Figure 1). These mutants provide distinct berry color phenotypes: *apricot, at*, loss of function in the isopentenyl diphosphate 1 (*ID11*) gene (Pankratov et al., 2016); *yellow flesh, r*, knockout of the *phytoene synthase 1* (*PSY1*) gene (Fray and Grierson, 1993); *tangerine, t*, loss of function in the carotenoid isomerase 1 (CrtISO1) enzymatic activity (Isaacson et al., 2002); *Beta, B*, and *Delta, Del*, gain of function in the *lycopene* β*-* and ε*-cyclase* (*CYC-b*; *LCY-e*) genes (Gil et al., 1999; Ronen et al., 2000); *high-pigment 3, hp3*, loss of function in the transcript coding for the *zeaxanthin epoxydase* (*ZEP*) (Galpaz et al., 2008); neoxanthin deficient 1, *nxd1*, defected in the neoxathin synthase (NXS) enzymatic activity (Neuman et al., 2014). In this context, the only known exception of a carotenoid structural gene which, if mutated, does not affect the berry color is represented by the β*-carotene hydroxylase 2* (*CHY2*), whose knock out produce the, so called, *white flower* (*wf*) mutant, displaying, respectively, regular and not pigmented fruits and flowers (Galpaz et al., 2006). Additionally, a series of well-known mutants in ABA biosynthesis are also available thanks to the studies carried out by the german scientist Hans Stubbe: *notabilis, not*, loss of function in the *9-cis-epoxycarotenoid dioxygenase* (*NCED*) gene (Burbidge et al., 1999); *flacca, flc*, knockout of the gene coding for a *molybdenum cofactor* (*MoCo*) (Sagi et al., 2002); *sitiens, sit*, deficient in the aldehyde oxidase (AAO) enzymatic activity (Harrison et al., 2011). More recently, the first mutant in the strigolactone pathway (*ORT1*) has been identified, although the source of the mutation has not yet been elucidated (Kohlen et al., 2012) (Figure 1). In addition, The *Solanaceae* genome network (SGN) and the Tomato Genetic Resource Center (TGRC) host large collections of tomato genotypes and mutants, which are available to researchers (Di Matteo et al., 2011; Saito et al., 2011; Bauchet and Causse, 2012; Sacco et al., 2013). More recently, a collection of ethyl methanesulfonate (EMS) and γ-ray-derived tomato mutants in the Micro-Tom dwarf background has been generated (Saito et al., 2011; Shikata et al., 2016). To date, it comprises over a thousand genotypes which have been used to create the TOMATOMA database, representing an interesting resource to research scored traits/phenotypes easily. Other EMS tomato mutant collections include the M82 processing tomato collection (http://zamir.sgn.cornell.edu/mutants/) and the Red Setter collection (http://www.agrobios.it/tilling/). These monogenic mutant populations could be directly screened to identify the genes responsible for a specific function (Menda et al., 2004; Long et al., 2006), or individual mutant lines could be analyzed to confirm the function of a gene previously identified by other means, such as QTL analysis (Goldsbrough et al., 1994).

**Figure 2:**
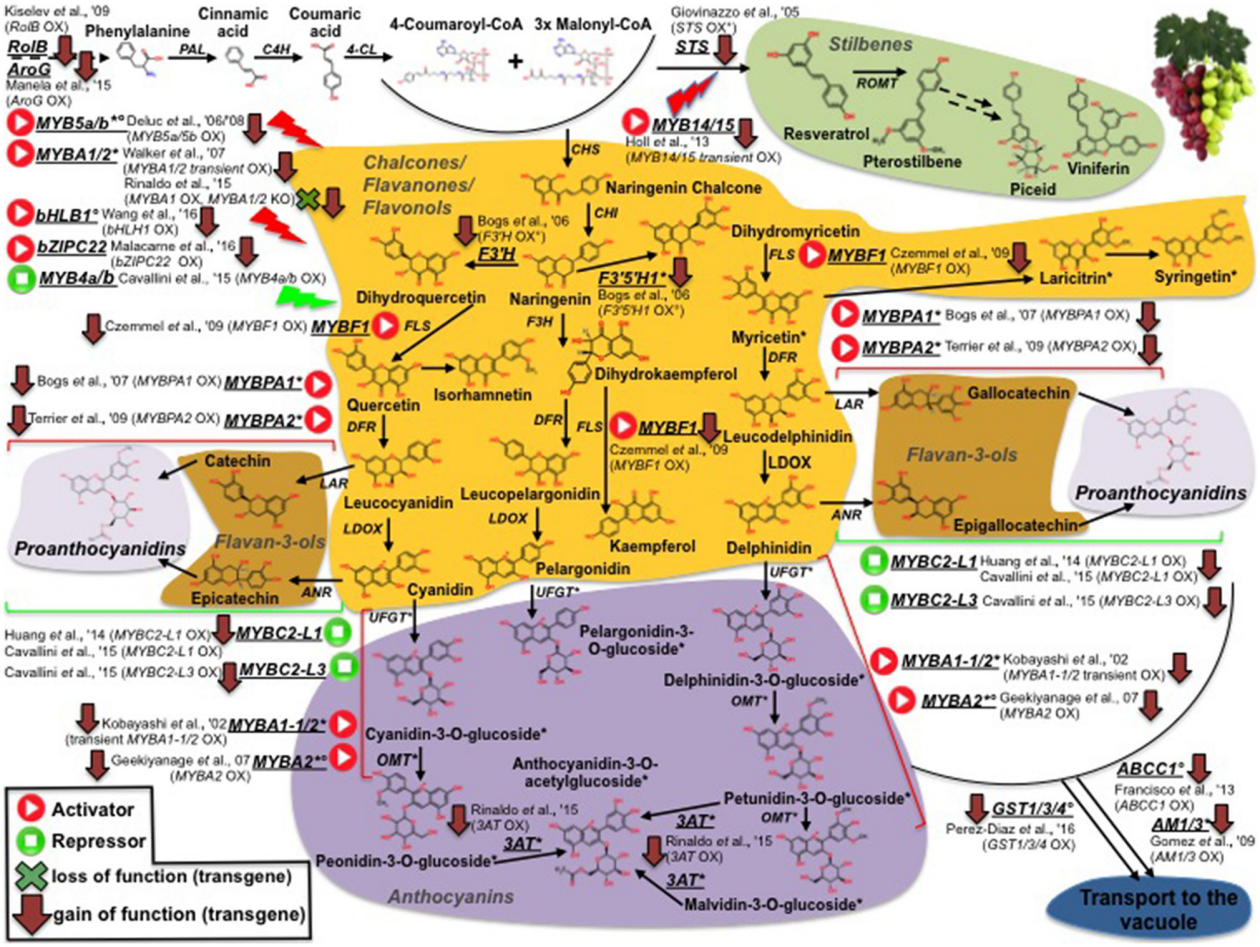
**Phenylpropanoid biosynthesis in grapevine and survey of structural and regulative genes engineered by genetic modification (GM)**. Transcription factors positively (activators) or negatively (repressors) affecting phenylpropanoid pathway are represented by “Play” and “Stop” symbols, respectively. Red arrows and green crosses pinpoint overexpression and knockout GM interventions, respectively. Asterisks indicate genes and metabolites previously reported to be not/low expressed and accumulated, respectively, in white genotypes. Degrees refer to ME studies in which grapevine genes were ectopically expressed only in heterologous systems.

Unlike tomato, collections of grapevine-induced mutants are quite rare (Fortes et al., 2015). Consequently, almost all studies in grape aimed at deciphering the molecular basis of traits use natural mutants (This et al., 2006). The FAO/IAEA Mutant Variety Database (MVD) maintains a wide range of plant mutant cultivars including tomato and grapevine. In grape, the counterpart of the conspicuous tomato/carotenoid system is represented by the phenylpropanoid pathway and, more specifically, by the synthesis of high-value sub-classes of phenypropanoid compounds (anthocyanins, stilbenes etc). An overview of grape genes and genetic resources for important phenylpropanoids affecting fruit quality is shown in Figure 2. While, contrary to the situation in tomato, it is not possible to clearly define grapevine monogenic mutants, several studies have unraveled the genetic basis of the difference between red and white cultivars, which is mainly due to a group of *MYB* transcription factors (*MYBA1-1/2, MYBA2, MYB5a/b*), mutated in the latter and, thus, preventing anthocyanin synthesis (Kobayashi et al., 2002; Deluc et al., 2006, 2008; Walker et al., 2007; Rinaldo et al., 2015; Figure 2). Similarly, Rinaldo et al. (2015) have reported that the acylated-anthocyanin phenotype is associated to the expression of the *3AT* gene, coding for an *ANTHOCYANIN 3-O-GLUCOSIDE-6*″*-O-ACYLTRANSFERASE*, which is lacking in white cultivars, as well in some red varities as Pinot-Noir, that do not accumulate acylated anthocyanins. TILLING was also used to screen the tomato mutant database (Kurowska et al., 2011) for validation of gene function and as a source/tool for crop improvement (Minoia et al., 2010). Furthermore, it can also be applied to the identification of SNPs in spontaneous mutants (EcoTILLING) making it, thus, extremely useful in characterizing the variability present in germplasm banks (Mba, 2013).

### Genome and epigenome sequencing and genotyping methods

Genomic variations can be the result of SNPs, insertions/deletions (Indels), copy number variations (CNV), and presence absence variations (PAV); they are responsible for crop evolution and domestication (Xu and Bai, 2015). Historically, to decipher genomic diversity, two types of molecular markers were developed (reviewed by Yang et al., 2015). The first were generated before the genomic era and were able to identify genetic diversity in a wide range of genotypes (and different conditions) without the need for DNA or genome sequences. For example, the first markers developed in the 1980s were the restriction fragment length polymorphism (RFLP). Anonymous PCR-based markers such as Random Amplified Polymorphic DNA (RAPD) markers and Amplified fragment length polymorphism (AFLP) were developed later. Single Sequence Repeat (SSR) or microsatellite markers were more popular during the 1990s and the early 2000s, when a large source of reliable medium-throughput markers was generated. However, even with these markers, molecular mapping remained time-consuming, expensive, and yielded relatively low mapping resolution (Xu and Bai, 2015). While several QTLs were identified on large genomic regions, few have been used in breeding programs (Bernardo, 2008).

Three generations of sequencing technologies resulting in three “waves” of genome sequencing facilitated the study of germplasm diversity and, thus, the production of new markers and high-throughput genotyping technologies that impact on breeding methods (Bolger et al., 2014; Varshney et al., 2014; Xu and Bai, 2015; Yang et al., 2015). In 2007, the genomes of an inbred line (PN40024) derived from Pinot Noir (Jaillon et al., 2007) and a heterozygous genotype nowadays used by winemakers (Velasco et al., 2007), were published by two groups independently. Both studies used whole genome shotgun (WGS) methods and predicted around 30 k protein-coding genes (Jaillon et al., 2007; Velasco et al., 2007) distributed around 38 chromosomes (*n* = 19). On the other side, a high quality, well-annotated reference genome is available for tomato (Sato et al., 2012). From this genome (around 900 Mb divided up to 12 chromosomes), 34,727 protein-coding genes were identified and 30,855 of these were confirmed by RNA sequencing. Moreover, using comparative genomics with grape and *A. thaliana* genomes, this study highlighted that two consecutive genome triplication events might have occurred during its evolution (Sato et al., 2012). The use of NGS methods is not limited to sequencing and *de novo* assembly but, thanks to an increase in high-throughput read lengths, single-base accuracy, reduced costs, and assembling methods, NGS enables whole-genome resequencing to identify genetic variations on a genome-wide basis (Xu and Bai, 2015). A number of resequencing projects have already identified genomic variations by resequencing and identifying a huge number of DNA markers (cited above). Divergence between the wild (*S. pimpinellifolium*) and domesticated tomato genomes was estimated at around 0.6%, representing 5.4 million SNPs, distributed along the chromosomes mostly in the gene-poor regions (Sato et al., 2012). Despite this, more than 12,500 genes carry non-synonymous changes. Another study has revealed that the Micro-Tom genome presents about 1,230,000 SNPs and 190,000 indels, by comparison with the “Heinz 1706” genome (Aoki et al., 2013). Using a high-density polymorphism array (7,720 SNPs, also known as the SolCAP array), Sim et al. (2012) genotyped a collection of 426 tomato accessions, which revealed that over 97% of the markers in the collection were polymorphic. Currently, several hundred resequenced genomes of tomato varieties, *S. lycopersicum* vr cerasiformes, and *S. pimpinellifolium* are available for marker and variability studies at https://solgenomics.net/jbrowse_solgenomics/. They are being used to gain an understanding of genetic base domestication and improvement, and for GWAS (Lin et al., 2014). WGS of induced tomato mutants reveals many DNA markers, such as SNPs (Menda et al., 2004; Saito et al., 2011; Xu and Bai, 2015). In some cases, NGS can be applied to a limited number of sites in the genome and the throughput can be increased using Genotyping By Sequencing (GBS) (Kumar and Khurana, 2014; Xu and Bai, 2015). For example, Víquez-Zamora et al. (2014) used GBS on a RIL population of a cross between *S. lycopersicum* cv. Moneymaker and *S. pimpinellifolium* G1.1554 to develop a linkage map of 715 unique genetic loci from 1,974 SNPs. These results were subsequently used to map QTL responsible for TYLCV (Tomato yellow leaf curl virus) resistance. A similar strategy based on the SolCAP was used by Rambla et al. (2017a) to define a volatile QTL map in an RIL population derived from the cross between *S. lycopersicum* (Money maker) and the TO-937 accession of *S. pimpinellifolium*.

Recent studies have shown the differential regulation of genes encoding epigenetic regulators as well as local chromatin and DNA methylation changes in response to a variety of abiotic stresses including cold, salinity, drought, osmolality, or mineral nutrition (reviewed by Fortes and Gallusci, 2017). Epigenetics constitutes another process that greatly influences gene expression and, therefore, contributes to genetic plasticity. DNA methylation represents a layer of regulatory complexity beyond that encoded in the basic structure of the plant genome (Harrigan et al., 2007). Using techniques such as bisulfite Sanger sequencing, whole-genome bisulfite sequencing, and chromatin immunoprecipitation sequencing (ChIP-seq), Zhong et al. (2013) have shown that tomato ripening involves specific epigenetic remodeling. They found that binding sites for *RIN*, one of the key ripening transcription factors, were frequently localized in the demethylated regions of the promoters of numerous ripening genes. This binding process occurred in concert with demethylation. The binding of *RIN* to regulate fruit ripening genes is attenuated in the *cnr* ripening mutant. In addition, they found that DNA regions near the 5′ ends of genes were hypermethylated in the *cnr* mutant (Zhong et al., 2013). In a more recent study (Liu et al., 2015), a direct relationship between DNA demethylase (*SlDML2*) activity and tomato fruit ripening was reported. Briefly, silencing *SlDML2* caused ripening inhibition *via* hypermethylation. Simultaneously, a drastic reduction in the expression of both transcription factors controlling fruit ripening and of down-stream pathways (e.g., carotenoids) occurred. Consequently, crop-improvement strategies should take account of both DNA sequence variation between plant lines and information encoded in the epigenome. In this context, the grape was recently proposed as an essential model for epigenetic and epigenomic studies in agriculturally-important, woody perennials to enable so-called epigenetic breeding (Fortes and Gallusci, 2017). Currently, a Tomato Epigenome database (http://ted.bti.cornell.edu/epigenome/1196099620) is available to investigate the presence of DNA methylation phenomena for each tomato gene. Epigenetic mechanisms have also been reported as being involved in defining the levels of Vitamin E accumulation in tomato fruits (Quadrana et al., 2014). Epigenetic marks may participate in the priming mechanisms to better withstand biotic and abiotic stresses, a topic that deserves attention in order to moderate stress susceptibility and increase climate change resilience in grapevine and tomato (reviewed by Fortes and Gallusci, 2017).

### Phenomics

While sequencing and genotyping technologies have leaped forward significantly, limited progress in the throughput and price affordability of phenotyping technologies has slowed the identification of genetic-phenotypic associations (Fiorani and Schurr, 2013; Bolger et al., 2014).

Phenotype-based selection came long before DNA discovery and the use of genotyping technologies. However, sequencing and molecular biotechnologies made rapid progress while phenotyping biotechnologies still need to be improved. Indeed, while the sequence of genonic DNA gives a comprehensive view of genetic capacity, the information it contains is cryptic and does not directly explain the differences between cells and all plant phenotypes (Angel et al., 2012). When it comes to fruit such as tomato or grape, it is the phenotype that is directly linked to our interest. Until now, plant phenotyping mainly focused on a single scale (molecule, cell, organ, plant, field, or canopy) depending on the organ of interest (shoots or roots) and the technology used. However, Rousseau et al. (2015) insist on the importance of multi-scale analysis. Indeed, genome expression can be observed at multiple microscopic and macroscopic levels including proteomics, metabolomics, physiological traits, and others that are visible/invisible to the naked eye. Hence, phenotypic traits provide more direct information about plant production and health than genomic data do. Nevertheless, because few technologies are available, phenotyping methods have traditionally been restricted to macroscopic traits.

Fortunately, the recent improvement in phenotyping methods (reviewed by Fiorani and Schurr, 2013) enable us to broaden the concept of phenotyping and include both molecular mechanisms (proteomic and metabolomic) and all intermediate layers that result in macroscopic physiological and phenological traits (architecture, yield, taste). Progress is mainly related to the development of a wide range of sensors, their automatization and adaptation to both indoor and outdoor conditions. Hence, advances in phenotyping technologies, including cost reductions and time gains, facilitate an increase in throughput phenotyping for multi-level traits under control or field conditions (reviewed by Fiorani and Schurr, 2013; Araus and Cairns, 2014). In fact, the global phenotype can be considered the result of all the measurable traits, influenced in a complex and dynamic manner (time and space) by both genome expression and environmental effects.

Macroscopic shoot phenotyping improvements have mainly been due to the development of new sensors (Table 4) (Araus and Cairns, 2014; Fahlgren et al., 2015). For root phenotyping, new technologies were recently established (Wasson et al., 2012; Fiorani and Schurr, 2013; Kuijken et al., 2015) by easily accessing the roots (artificial growth medium and dynamic 2D or 3D imaging), and by indirect methods which phenotype roots in the soil (Table 5). For example, using a time-lapse scanning system, Dresbøll et al. (2013) demonstrated that the growth rate of tomato roots decreased under waterlogging. More recently, a series of platforms that integrate morphological parameters and, in some cases, gene expression have been developed. Among these, for example, MorphoGraphX is able to quantify several morphogenetic processes in 4D (Barbier de Reuille et al., 2015). New sensors were also developed to improve post harvested practices such as shelf life (Abano and Buah, 2014). For example, NIR spectroscopy was used to optimize the storage time of apple lots (Giovanelli et al., 2014).

**Table 4:** **New sensors and their application to plant macroscopic phenotyping**.

**Sensor technology**	**Measure**	**Applications**	**References**
Sensitive cameras in the visible spectral range of the electromagnetic spectrum.	Produce raw data in the RGB or in the HSV (hue, saturation, value) spaces.	Shoot phenology and color.	Fiorani and Schurr, 2013; Araus and Cairns, 2014
Fluorescence cameras.	Analysis of fluorescence parameters.	Photosynthesis status.	Maxwell and Johnson, 2000; Berger et al., 2004; Bélanger et al., 2008; Chaerle et al., 2009; Fiorani et al., 2012; Fiorani and Schurr, 2013; Araus and Cairns, 2014
		Identification of biotic and abiotic stresses before visible phenotypes could be detected.	
Thermal cameras.	Measure the leaf temperature.	Identification of abiotic (Fuentes et al., 2012; Mishra et al., 2012), and biotic (Calderón et al., 2014; Raza et al., 2015) stresses.	Review by (Fiorani and Schurr, 2013; Meron et al., 2013; Araus and Cairns, 2014; Calderón et al., 2014; Prashar and Jones, 2014)
		Evaluation of fruit maturity and bruise (Vadivambal and Jayas, 2011; Ishimwe et al., 2014).	
Imaging spectroscopy.	Scanning specific wavebands of interest through high resolution cameras.	Water status by the analysis of the Near-Infrared (NIR) to the mid-infrared wavebands.	Fiorani and Schurr, 2013; Giovanelli et al., 2014
		Photosynthesis status by the analysis of the peak of green reflectance at 550 nm.	
		Determination of nitrogen content and pigment composition (Fiorani and Schurr, 2013).	
		Estimation of storage time for apple using NIR.	
I-sensor.	Measurement of electrical impedance.	Estimation of cuticule and wax characteristics on vine berries and the link with disease resistance.	Herzog et al., 2015

**Table 5:** **3D imaging technology for plant phenotyping**.

**3D sensor**	**Measures**	**Application**
Stereo camera.	3D imaging.	Biomass and shoot structure.
High resolution volumetric imaging (X-ray tomographs, Magnetic resonance imaging, and positron emission detectors).	3D imaging of physiological status.	Water content, morphometricparameters.
Laser scanning technologies such as Light Detection And Range (LIDAR) (Menzel et al., 2009; Eitel et al., 2011; Hosoi and Omasa, 2012; Araus and Cairns, 2014; Deery et al., 2014; Raza et al., 2015; Rousseau et al., 2015).	Measures the distance between a target and the sensor by analyzing the reflected light of a laser.	Canopy characterization such as phenology, and leaf area index (Llorens et al., 2011; Rinaldi et al., 2013; Sanz et al., 2013; Hosoi et al., 2011).

On the other hand, automated facilities have evolved into high-throughput phenotyping platforms providing a powerful tool to fundamental research that can be conducted at growth chamber, greenhouse or field levels. In order to reduce error variance under field conditions, most of the sensors described above could be adapted to allow high-throughput measures, thus increasing the number of samples under analysis (reviewed by Araus and Cairns, 2014). Ground vehicles equipped with sensors were used in several studies (Andrade-Sanchez et al., 2014), while aerial vehicles with dedicated instruments facilitate rapid plant characterization in many plots, notably for phenotyping canopy traits (Araus and Cairns, 2014; Sankaran et al., 2015). Among them, due to their reduced cost, user-friendly flying control, and high autonomy, polycopters also called Unmanned Aerial Platforms (UAPs) could constitute the future of field phenotyping. The laboratory of plant-microbe interactions (INRA, Toulouse, France) set up a low cost phenotyping platform so called “Heliaphen,” which allows the growth and the high throughput phenotyping of 1,300 plants in outdoor semi-natural conditions (https://www.youtube.com/watch?v=VZSvgeWuhlw). The development of plants in high capacity pots (15 L) makes possible the study of crops during their entire life cycle. In this way, the effect of soil heterogeneity is reduced compared to field conditions. The use of a mobile robot, which phenotypes and monitors hydric conditions for each plant, is one of the original aspects of this platform (personal communication from N. Langlade).

In microscopic imaging technologies, improvements in time acquisition, automatization, and user-friendly interface make high-throughput phenotyping possible on a microscopic scale (Sozzani et al., 2014; Rousseau et al., 2015). In a recent study, Legland et al. (2012) coupled microscopic and macroscopic approaches to create a synthetic representation of cell morphology variations at the whole fruit level. The complexity and the high volume of data produced by high-throughput phenotyping platforms require computing power and robust bioinformatic tools (Araus and Cairns, 2014). Furthermore, to date, phenotyping data are still dispersed in different file types, programs, and databases and, therefore, efforts to comply with defined standards, which enable comparison and information exchange between phenotyping experiments and conditions, are needed (Krajewski et al., 2015).

### Proteomics

The proteome integrates environmental and genetic information and is, therefore, fundamental. Knowledge of the proteome permits a more direct connection between proteins and the corresponding phenotypes (Boggess et al., 2013). Nowadays, significant improvements have been achieved in this field (reviewed by Angel et al., 2012). For example, coupling liquid chromatography (LC) separations with mass spectrometry (MS)-based technologies that enable the characterization of a protein at the proteome and sub-proteome levels, such as post-translational modifications (PTMs) of proteins like, for instance, lysine succinylation (Jin and Wu, 2016). Hence, many studies have used proteomic analyses to highlight the link between proteomic and phenotypic variations (Tanou et al., 2009; Zhao et al., 2013; Kumar and Khurana, 2014). Several studies of tomato proteome have provided both qualitative and quantitative data (reviewed by Kumar and Khurana, 2014). For example, using shotgun proteomic analysis of fruit tissues, Shah et al. (2012) presented data about the interaction between tomato fruit and *Botrytis cinerea* showing that the proteins produced by the fungus include those that facilitate the pathogen's penetration and growth on the plant tissue, those that inhibit resistance responses by the plant, and those that enable the pathogen to use the nutrient resources within the plant. On the other hand, the proteins produced by the plant include those that limit pathogenic infection and protect the plant tissue from additional damage.

A similar study by (Parker et al., 2013) analyzed the interaction between tomato and the *Pseudomonas syringae* bacteria through an iTRAQ (isobaric tags for relative and absolute quantification) quantitative proteomic approach. Proteomic data could also be used as biomarkers to facilitate the rapid identification of biotic or abiotic stress before it becomes visible through diagnostic tools (Angel et al., 2012). An interesting, novel approach involves the use of combined genomic-proteomic data to predict DNA-binding proteins (like transcription factors), integrated through computational models which can greatly promote functional annotation of tomato or other plant genomes (Motion et al., 2015). However, in contrast to the genomic data common to all cells of the same organism, proteomic data could be highly tissue-, cell-, or compartment-specific, making it more difficult to access the overview offered by plant proteome. In this context, another important issue is represented by the characterization of the protein fraction at sub-cellular level, like those specifically synthesized in plastids (Barsan et al., 2012), which can significantly influence a series of physiological processes such as fruit ripening. In another example, the characterization of proteomic changes induced during ripening processes into grape fruit skin provided important information to determine the skin parameters which could impact on wine quality (Deytieux et al., 2007). Furthermore, alterations in sugar and phenylpropanoid metabolism due to thermal stress were revealed by a quantitative proteomic study of Cabernet Sauvignon grape cells (George et al., 2015).

### Metabolomics

Metabolomics has played a remarkable role in assessing genotypic and phenotypic diversity in plants, in defining biochemical changes associated with developmental changes during plant growth and, increasingly, in compositional comparisons. Furthermore, metabolic information is often viewed as a more accurate reflection of biological endpoints than transcript or protein analysis (Harrigan et al., 2007). Therefore, metabolomic data may strongly support breeding and selection of novel yield-enhanced and nutritionally improved crops (Harrigan et al., 2007). It also seems that metabolite composition, although genetically based, is heavily influenced by environmental factors, much more, even, than enzyme activity (Biais et al., 2014). Reassuring results have proved that the hereditability of the tomato fruit metabolome, including that part of the metabolome affecting flavor, in terms of mQTL, was relatively high, in both primary metabolites (sugars and acids) (Schauer et al., 2008) and volatiles (Rambla et al., 2016). Obviously, flavor-related traits have attracted much attention. The combination of a taste panel and other omics technologies have facilitated the definition of sugars, organic acids, and volatile compounds underlying flavor and consumer preferences (Mathieu et al., 2009). Furthermore, the robustness of the mQTL and the release of flavor compounds often depend on enzymatic activities that cleave the chemical bond between the flavor compound and a glycosyl moiety. One example is represented by the *non-smoky glycosyltransferase1* (*NSGT1*) gene, that takes part in the phenylpropanoid pathway, which was shown to prevent the “smoky” aroma attribute (Tikunov et al., 2013). Similar glycosylation/glycosidation mechanisms operate in grape varieties that usually accumulate large amounts of volatile precursors as conjugated compounds that are released following tissue maceration (Rambla et al., 2016, 2017b). Using target approaches based on knowledge of metabolic pathways has led to the characterization of several genes involved in the biosynthesis of phenylpropanoids (Tieman et al., 2010; Mageroy et al., 2012), fatty acid-derived volatiles (Speirs et al., 1998; Chen et al., 2004; Matsui et al., 2007; Shen et al., 2014), apocarotenoids (Simkin et al., 2004), esters (Goulet et al., 2015), and other phenylalanine-derived volatile compounds (Tieman et al., 2010), and in the conjugation and/or deconjugation and emission of volatiles (Tikunov et al., 2013). Moreover, Schauer et al. (2005) performed one of the first GC–MS-based surveys of the relative metabolic levels of leaves and fruits of *S. lycopersicum* and five sexually-compatible wild tomato species (*S. pimpinellifolium, S. neorickii, S. chmielewskii, S. habrochaites*, and *S. pennellii*). Interestingly, several biochemical markers associated with the desired traits (stress resilience, nutritional quality) were identified in the wild species. A series of robust LC–MS-based protocols for tomato metabolome have been developed at WUR (De Vos et al., 2007) and KAZUSA (Iijima et al., 2008), and exploited in several studies of fruit development and physiology (Yin et al., 2010; Mounet et al., 2012), and stress response (Etalo et al., 2013; Lucatti et al., 2013). In a recent study (D'Esposito et al., 2017), genotype × environment interaction, particularly related to sensorial attributes, was investigated in three tomato varieties using a combination of genomic, transcriptomic and metabolomic technologies. The varieties in question included the “cosmopolitan” Heinz 1706—which showed high resilience in the different environments tested—and two Italian Protected Designation of Origin (DOP) ecotypes—San Marzano and Vesuviano—which displayed high plasticity to environmental variations.

In grape, studies focusing on ripening and using complementary platforms such as NMR and GC–MS to identify metabolic markers of pre-ripening and ripening stages, are available (Fortes et al., 2011; Agudelo-Romero et al., 2013). Using an integrated transcriptomic/metabolomic approach, Agudelo-Romero et al. (2013) provided hints about how the development of a grape cultivar-specific aroma is controlled at transcriptional level. In the same context, the distinctive processes regulating the accumulation of polyphenols in berry skins of Cabernet Sauvignon and Shiraz cultivars were investigated at gene expression and metabolite levels (Degu et al., 2014).

One important phenological aspect, the terroir (i.e., the complex of all environmental factors responsible for the qualities of a grapevine cultivar grown in a specific habitat), was studied for the Corvina variety using volatile/non-volatile metabolomics, and transcriptomics. On the whole, a strong terroir-specific effect was revealed in clones grown in different vineyards—an effect that persists over several vintages (Anesi et al., 2015). The primary aromatic profile of a wine is mainly due to the genotype × environment-derived relationship between volatile metabolites and their precursors. Volatiles have been extensively studied in grape (reviewed in: González-Barreiro et al., 2015), whereas volatile precursors have scarcely been investigated (Martin et al., 2012). Recently, Rambla et al. (2016) performed an in-depth analysis of volatile and precursor metabolites in white (Airén) and red (Tempranillo) grape variety berries at different developmental stages. The use of a series of bioinformatic approaches—such as correlation networks—proved the existence of complex metabolite-metabolite patterns that were more complex in Airén, as would be expected given the enriched aroma bouquet typical of white varieties. Metabolomics has contributed much to our increased understanding of the molecular basis of biotic stress resistance. A series of metabolites, including quercetin-3-O-glucoside and a *trans*-feruloyl derivative, have been shown to underlie cultivar resistance to downy mildew infection (Kashif et al., 2009). More recently, Agudelo-Romero et al. (2015) concluded that berries infected with *B. cinerea*, reprogram carbohydrate and lipid metabolisms toward increased synthesis of secondary metabolites like trans-resveratrol and gallic acid, which are involved in plant defense.

Furthermore, metabolomic approaches have been used to assess the impact on the metabolome and fruit quality traits of mutations or genetically engineered approaches in structural/regulatory genes. Of special significance are the metabolic boost identified in tomato fruit by the light-hyperresponsive *high-pigment* (*hp*) gene (Bino et al., 2005). The authors concluded that fruits from *hp* plants overproduced many metabolites with antioxidant or photoprotective activities. A number of additional tomato fruit color mutants that affect the metabolite profile have been identified (list available at http://kdcomm.net/~tomato/Tomato/color.html). However, not all of these them resulted in the accumulation of quality molecules (with positive health or organoleptic effects) in the fruit. Among these mutants are the *B* (*Beta*) and *B*^*c*^/*B*^o*g*^ mutants, yielding high amounts in β-carotene and lycopene, respectively, due to a gain or loss of function in chromoplast-specific lycopene β-cyclase (*Cyc-B*) activity (Ronen et al., 2000, and Figure 1). Similarly, the *Abg* (*Aubergine*), *Aft* (*Anthocyanin fruit*) and *Atv* (*Atroviolaceum*) loci result in anthocyanin-accumulating fruits (Mes et al., 2008; Schreiber et al., 2012), phenotypes associated with a perturbation in the expression of the transcription factors controlling anthocyanin synthesis, such as *ANTHOCYANIN 1* (*ANT1*) and *ANTHOCYANIN 2* (*AN2*). In contrast to classical mutants, metabolic engineering overcomes a number of classic breeding constraints, including a limited gene-pool, time consuming processes, etc. Against this broader scenario, tomato fruits have been engineered to accumulate large amounts of many high-value nutrients (in an approach known as metabolic engineering, ME): vitamins such as folate (Díaz de la Garza et al., 2007) and ascorbate (Nunes-Nesi et al., 2005); secondary metabolites such as carotenoids, for which tomato represents a model system. An overview of ME studies of carotenoids in tomato is shown in Figure 1: so far, transgenic fruits enriched in lycopene [(Fraser et al., 2002, 2007); (ectopic expression of the bacterial (*CrtB*) or the tomato (*PSY1*) *phytoene synthase* genes); (Rosati et al., 2000) (down-regulation by antisense technology, of the *lycopene-b-cyclase 1* (*LCY-b1*) gene)], β-carotene [(Apel and Bock, 2009); transplastomic expression of the bacterial lycopene-β-cyclase (CrtY) activity]; (D'Ambrosio et al., 2004, 2011) (stable transgenics for the tomato *LCY-b1* gene); (Römer et al., 2000) [ectopic expression of the bacterial *carotenoid isomerase* (*CrtI*); (Rosati et al., 2000) (stable transformants espressing the arabidopsis *LCY-b1* gene), lutein (Giorio et al., 2013; over-expression of the endogenous lycopene ε-cyclase (LCY-ε-) activity)], and β–xanthophylls [Dharmapuri et al., 2002; simultaneous expression of the arabidopsis *LCY-b1* and of a pepper β*-carotene hydroxylase 1* (*CHY1*)]; (D'Ambrosio et al., 2011) [overexpression of the tomato β*-carotene hydroxylase 2* (*CHY2*)] have been achieved. Furthermore, ME tomatoes accumulating high-value ketocarotenoids (e.g., astaxanthin) have been obtained by the simultaneous expression of the β*-carotene hydroxylase* (*CrtZ*) from *Haematococcus pluvialis* and the algal β*-carotene ketolase* (*CrtW*) from *Chlamydomonas reinhardtii* (Huang et al., 2013) (Figure 1). In some cases, it is not possible to achieve stable silenced transgenic plants for a specific activity, likely due to the occurrence of a lethal phenotype in the transformant cells; in this context, an useful alternative is represented by virus induced gene silencing (VIGS), which allows to study a specific enzymatic step by transient transformation assays. In tomato fruits, this tool has been exploited to investigate the functions of all the genes involved in lycopene biosynthesis (*PSY1, 2, 3*; phytoene desaturase, *PDS*; 15-*cis*-ζ-carotene isomerase, *Z-ISO*; ζ-carotene desaturase, *ZDS*; carotenoid isomerase 1, like-1, like-2,*CrtISO1, CrtISO-LIKE1, CrtISO-LIKE2*), and the presence of three functional units, comprising PSY1, PDS/ZISO, and ZDS/CrtISO has been found (Fantini et al., 2013). ME has also been used to elucidate enzymatic activities taking place in carotenoid catabolism: with this purpose, apocarotenoid emission has been strongly reduced by the down-regulation, *via* RNAi technology, of the *carotenoid cleavage dioxygenase 1b* (*CCD1b*) gene (Simkin et al., 2004). Similarly, ABA biosynthesis has been investigated by through the production of RNAi plants for the 9-*cis*-*epoxycarotenoid dioxygenase* (*NCED1*) gene (Sun et al., 2012); and two CCD (*CCD7* and *CCD8*) transcripts, involved in strigolactone pathway, have been characterized by tomato stable transformants, in which the two enzymatic functions had been knocked out (Vogel et al., 2010; Kohlen et al., 2012). Engineering tomatoes for high flavonoids in the fruit is a biotechnology goal as theise types of healthy metabolites are deficient in the fruit. To this end, successful efforts for flavonoid increase (Schijlen et al., 2006) and *de novo* anthocyanin accumulation (Zhang et al., 2013) have been reported; in a recent study, Zhang et al. (2015) used the *AtMYB12* transcription factor to engineer high levels of novel phenylpropanoids in tomato. This up-regulation of specific branches of phenylpropanoid metabolism was disclosed by a combination of RNA sequencing and LC–MS analyses. Phenylpropanoids have also been the target molecules of the few ME attempts reported in grape (illustrated in Figure 2): interestingly, while only limited studies have modified the expression of structural genes, most efforts have focused on the identification of biosynthetic transcriptional regulators. Within the formers, *flavonoid 3*′*-hydroxylase* (*F3*′*H*) and *flavonoid 3*′*,5*′*-hydroxylase* (*F3*′*5*′*H*), key genes for flavonoid hydroxylation (and, thus, for their stability, color and antioxidant capacity) have been cloned in red grapevine, cv Shiraz, and their functionality has been proved by ectopic expression in *Petunia hybrida* (Bogs et al., 2006); in another study, Giovinazzo et al. (2005) have achieved stilbene accumulation in tomato fruits by expressing a grape *stilbene synthase* (*STS*). In the latter, a vast range of *MYB* transcription factors acting as activators or repressors of the pathway have has been described: interestingly, some of them have been found to perturb the whole biosynthesis [positively: *MYBA1-1/2, MYBA2, MYB5a/b* (Kobayashi et al., 2002; Deluc et al., 2006, 2008; Walker et al., 2007; Rinaldo et al., 2015); negatively: *MYB4a/b* (Cavallini et al., 2015)], while another group looks to affect distinct phenylpropanoid sub-classes [*MYB14/15*, directly activating *STS* genes (*STSs*)] (Höll et al., 2013); *MYBF1*, positively regulating *flavonol synthase* (*FLS*) expression (Czemmel et al., 2009); *MYBPA1/2* and *MYBC2-L1/3*, respectively boosting or repressing flavan-3-ols/ proanthocyanidin synthesis (Bogs et al., 2007; Cavallini et al., 2015; Figure 2). Besides MYBs, additional transcription factors affecting phenylpropanoid metabolite pool have been isolated and characterized in grape: Wang et al. (2016), for instance, have identified a *VvbHLH1* factor, whose ectopic expression in Arabidopsis resulted in increased flavonoid content, although this factor looks to be also associated to ABA-related processes, like drought and salt stresses; similarly, Malacarne et al. (2016) have recently described a new bZIP factor, named *VvibZIPC22*, whose ectopic expression in tobacco has proved its role in triggering flavonoid synthesis and accumulation (Figure 2). Once synthesized, flavonoids and anthocyanins are rapidly transported to the vacuole: basically, three mechanisms including vesicle trafficking, membrane transporters and glutathione *S*-transferase (GST)-mediated transport have been described. In grape, in particular, two kinds of anthocyanin active transporters, and localized to the tonoplast, have been discovered: two belonging to the Multidrug And Toxic Extrusion (MATE) family and called *anthoMATE1-3* (*AM1* and *AM3*), which can bind acylated anthocyanins and translocate them to the vacuole in the presence of MgATP (Gomez et al., 2009); and an ABC-type transporter, *ABCC1*, shown to perform the transport of glucosylated anthocyanidins (Francisco et al., 2013). More recently, three GSTs (*VviGST1, VviGST3, VviGST4*) have been tested for their ability to bind glutathione and monomers of different phenylpropanoids (anthocyanin, PAs, and flavonols): interestingly, all the three genes displayed the binding activity, although with distinct specificity according the phenylpropanoid class (Pérez-Díaz et al., 2016).

## How knowledge of the genetic basis of the observed variability could contribute to improve fruit quality

Over the last 25 years, a number of papers have started to dissect the genetic basis of fruit quality traits by means of QTL analysis (Duchêne et al., 2012; Klee and Tieman, 2013). In tomato, fruit morphology, yield, fruit color, and soluble solid concentration were the major focus of attention during the early QTL mapping years but recently, more complex traits such as primary metabolites, nutritional, antioxidant, and volatile compounds have received more attention (reviewed by Grandillo et al., 2013; see Table 6). The translation of those early studies into gene discovery and/or application to breeding programs remains slow. This low impact can be explained in several ways, including the limited accuracy of QTL mapping experiments due to the lack of sufficient markers; the accuracy of phenotypic evaluations; or the limitations or poor suitability of mapping population designs (Collard et al., 2005), among others.

**Table 6:** **QTL analysis in tomato and grape**.

**Species**	**QTL or Candidate Genes (CG)**	**Characters**	**References**
Tomato	QTL and CG	Tolerance to chilling	Oyanedel et al., 2001; Elizondo and Oyanedel, 2011; Arms et al., 2015
Tomato	QTL	Shot turgor maintenance	Truco et al., 2000
Tomato	QTL	Flavor and gustative quality of berries	Saliba-Colombani et al., 2001; Causse et al., 2004; Schauer et al., 2006; Tieman et al., 2006; Mathieu et al., 2009; Zanor et al., 2009; Zhang et al., 2015; Calafiore et al., 2016
Tomato	QTL	Flowering characteristics	Tanksley et al., 1996; Doganlar et al., 2002; Jiménez-Gómez et al., 2007; Nakano et al., 2016
Tomato	QTL	Fruit Morphology, color, soluble solid concentration, yield	Eshed and Zamir, 1995; Fulton et al., 1997; Bernacchi et al., 1998; Saliba-Colombani et al., 2001; Monforte et al., 2001; Van der Knaap et al., 2002; Gur and Zamir, 2004; Huang and van der Knaap, 2011
Tomato	QTL	Carotene/nutritional/vitamins	Saliba-Colombani et al., 2001; Liu et al., 2003; Rousseaux et al., 2005; Schauer et al., 2006; Capel et al., 2015
Grape	QTL	Disease resistance	Fischer et al., 2004; Marguerit et al., 2009; Riaz et al., 2006, 2011
Grape	CG	Disease resistance	Barker et al., 2005; Coleman et al., 2009; Barba et al., 2014; Feechan et al., 2013
Grape	QTL	Pest resistance	Doucleff et al., 2004; Fischer et al., 2004; Zyprian et al., 2016; Krivanek et al., 2006; Xu et al., 2008
Grape	QTL		

In spite of these shortcomings, genes involved in tomato fruit morphology and sugar content QTLs have been isolated (Fridman et al., 2000; Monforte et al., 2014). Recent advances in sequencing, genotyping, and phenotyping technologies, combined with the development of a wide range of plant germplasm collections and populations, facilitate more accurate QTL detection (Chen et al., 2015; Li and Sillanpää, 2015). Today, these technologies permit the fine mapping of QTLs and candidate genes for a wide range of complex traits such as seed characteristics (Doligez et al., 2013), developmental stages (Duchêne et al., 2012), or tolerance to root chilling (Arms et al., 2015). In this last study, Arms et al. (2015) took advantage of a sub-NILs population in order to identify and functionally test candidate genes. Recently, Houel et al. (2015) worked on QTLs related to leaf area and berry quality using high-throughput genotyping technology from the Illumina® 18 K SNP chip and a mapping population of 129 microvines derived from Picovine × Ugni Blanc flb. The compact size, early flowering, and continuous production of reproductive organs make the Microvine or Dwarf and Rapid Cycling and Flowering (DRCF) mutant a valuable tool for QTL mapping (Houel et al., 2015). Combined with the 6,000 SNP markers given by the 18 K SNP chip, this microvine population has facilitated the identification of 10 QTLs of the 43 traits analyzed simultaneously (Houel et al., 2015). In tomato, the development of the Illumina® 8 K SNP chip (Sim et al., 2012) gave the research community access to affordable high-throughput genotyping. The combination of bulk segregant analysis with whole genome sequencing (i.e., QTL-seq) is another approach that has proved a cost-effective method of identifying QTLs involved in tomato fruit morphology (Illa-Berenguer et al., 2015).

Hence, several studies insist on the importance of the populations used to permit QTL fine mapping (Nicolas et al., 2016). Indeed, the choice of an appropriate genotype panel from the vast germplasm available is particularly relevant for QTL identification either in the case of using a segregating mapping population (Table 2) or in GWASenome Wide Association Studies (GWAS). Take, for example, one of the biggest collection of grapevine cultivars: that of the Institut National de la Recherche Agronomique (France). The 2,486 unique grapevine cultivars in this collection can be used to identify new QTLs (Nicolas et al., 2016). From this huge population, Nicolas et al. (2016) designed a diversity core panel of 247 grapevine cultivars with limited relatedness to use in identifying new QTLs with the GWAS approach as it captures most of the genetic and phenotypic diversity present in the original collection. Even though GWAS is a very promising strategy, the development of bi-/multi-parent populations is still highly relevant (Pascual et al., 2016) when comparing QTL detection in tomato RIL, MAGIC populations and GWAS, to find significant differences between the populations. RILs and MAGICs are especially powerful tools for rare allele mappings, whereas GWAS provides a more general view of common variants. An integration of different populations and mega QTL analysis (Monforte et al., 2014), would help detect an increasing number of small effect loci. High-throughput genotyping methods also help speed up the construction of time-consuming populations as IL collections (Barrantes et al., 2014). We would encourage the development of a larger number of these populations (especially ILs and MAGICs/NAMs) in the near future, to allow easy access to a wide range of germplasm resources.

One critical issue following QTL identification is to determine the stability and robustness of their genetic basis in different backgrounds and environments. Several studies have addressed the stability of QTLs over time and generation, as well as across environments (Monforte et al., 2001; Gur and Zamir, 2004; Chaïb et al., 2006; Doligez et al., 2013; Arms et al., 2015; Houel et al., 2015). These authors have shown that selecting stable QTLs to introgress into agronomic cultivars is feasible, a finding that must especially be taken into account considering issues relating to global warming. Introgression lines have been proved to be a highly suitable population design to address these questions (Monforte et al., 2001; Gur and Zamir, 2004).

Quantitative trait loci maps have been published for most descriptors of tomato fruit quality (color, texture, flavor) and also for specific metabolites associated with these quality descriptors. For these tomato fruit volatiles, QTLs have been identified in experimental populations obtained from crosses between tomato cultivars and different germplasm sources used as donor parents—e.g., cherry tomato (Saliba-Colombani et al., 2001; Zanor et al., 2009) or the distantly related, green-fruited, wild tomato species *Solanum pennellii* (Tadmor et al., 2002; Tieman et al., 2006) and *Solanum habrochaites* (Mathieu et al., 2009). In some cases, QTL validity (Zanor et al., 2009; Rambla et al., 2016, 2017a) has been confirmed in other populations which are, therefore, useful for breeding. Genomics has been successfully used in a limited number of cases to narrow down the regions of several hundreds of genes to a plausible candidate gene, as in the aforementioned case of the “smoky” aroma (Tikunov et al., 2013), and the gene for Brix (Zanor et al., 2009). In most cases, however, the gene underlying the QLT has yet to be identified.

## New plant breeding techniques (NPBT) for fruit quality studies

Over the past 10 years, the introduction of so-called, new plant breeding techniques (NPBT) has constituted a breakthrough in the field of crop improvement. A number of technologies have been developed to produce new plants with desired traits, in which the main bottlenecks to standard genetic modification (i.e., the presence of foreign DNA in the modified food plant) are no longer an issue. In this context, several different strategies, based on the exploitation of chimeric nucleases have been applied. Overall, they rely on a system composed of sequence-specific DNA-binding domains coupled to a non-specific DNA cleavage module (reviewed in: Gaj, 2014; Sprink et al., 2015; Schaart et al., 2016) that expedite efficient genomic modifications through the introduction of sequenced specific/targeted DNA double-strand breaks (DSBs), which boost all the DNA repair components, like error-prone non-homologous end joining (NHEJ), and homology-directed repair (HDR). To date, the most widely utilized NPBTs are: zinc finger nucleases, ZFNs; transcription activator-like effector nucleases, TALENs; and Clustered Regulatory Interspaced Short Palindromic Repeats (CRISPR)/CRISPR-associated (Cas) system, CRISPR/Cas. Each strategy has its own advantages and disadvantages, as illustrated in Table 7. To date, no TALEN and ZNF studies of grape are available, whereas two proof-of-concept trials have been described in tomato: Lor et al. (2014) knocked out the *PROCERA* (*PRO*) gene involved in the negative regulation of gibberellin signaling; in contrast, Hilioti et al. (2016) have shown the effectiveness of the ZFN approach by targeting the expression of the *LEAFY-COTYLEDON1-LIKE4 (L1L4)* transcription factor, coding for the β subunit of nuclear factor Y and severely affecting plant development.

**Table 7:** **Overview of the three main strategies for plant gene editing: ZFN, TALEN, and CRISPR/Cas9**.

**Technology**	**Acronym**	**Type**	**System components**	**Mechanism of action**	**Sensitivity to methylation**	**Off-target effects**	**Design difficulty**	**Scaling up for library production**	**Studies in tomato and grape**
Zinc finger nuclease	ZFN	Protein-DNA	Zinc finger DNA binding domain fused with an endonuclease (usually FokI); specific recognition of 3 bp sequences	A DNA-cutting/DNA-grabbing-based system, able to recognize target genes	Yes	High	Difficult	No (custom protein selection for each gene)	Hilioti et al., 2016
Transcription activator-like effector nuclease	TALEN	Protein-DNA	Endonuclease (usually FokI) catalytic domain fused to Xanthomonas spp. DNA binding domain of transcription activator-like effectors. Composed by 33–35 amino acid (aa) multiple repeats containing a repeat variable diresidue (RDV; usually, the aa 12 and 13)	Same as ZFN	Yes	Low	Medium	Possible but complicate	Lor et al., 2014
Clustered regularly-interspaced short palindromic repeat/CRISPR-associated	CRISPR/Cas9	RNA-DNA	20 nt crRNA fused to tracrRNA and Cas9 endonuclease	A DNA-cutting protein associated to a guided RNA which can specifically recognize target genes	No	Low	Easy	Easy (generation of 20 nt adapter/s for each gene)	Brooks et al., 2014; Ron et al., 2014; Čermák et al., 2015; de Toledo Thomazella et al., 2016; Jacobs and Martin, 2016; Ito et al., 2015; Klap et al., 2016; Pan et al., 2016; Xu et al., 2016; Ren et al., 2016; Wang et al., 2016

*Technical characteristics and a survey of all the studies described, to date, in tomato and grape are also provided*.

Currently, the most promising NPBT is based on the exploitation of the CRISPR/Cas9 system. Involved in the immune response processes of the prokaryotes (Barrangou et al., 2007), CRISPRs have been identified in 90% of sequenced archaea (Grissa et al., 2007). A simplified CRISPR system, relying on a single protein (Cas9), has been shown capable of modulating expression of specific one-by-one targets in human cells, insects and plants (Shalem et al., 2014; Konermann et al., 2015). More recently, a powerful tool for multi-modular expression of several plant genes in a single construct (with so-called “Goldenbraid” technology; Sarrion-Perdigones et al., 2011, 2013) has been adapted to CRISPR/Cas9 technology to build constructs able to modify the expression of a series of targets of interest (Vazquez-Vilar et al., 2016). Examples of efficient modifications of specific target genes have been reported both for tomato and grape: by using the CRISPR/Cas9 system. In fact, the *ripening inhibitor* (*RIN*) gene, encoding a MADS-box transcription factor regulating ethylene synthesis and, thus, fruit ripening, has been successfully mutagenized (Ito et al., 2015); simultaneously, the efficient knockout of the *L-idonate dehydrogenase* gene (*IdnDH*), involved in the tartaric acid pathway, has been achieved in both grape cell suspension and plants (Ren et al., 2016). Additionally, still in grape, a computational survey of all the CRISPR/Cas9 sites available in the genome has been performed. This has revealed the presence of 35,767,960 potential CRISPR/Cas9 target sites, distributed across all chromosomes with a preferential localization at the coding region level (Wang et al., 2016). A Grape-CRISPR website of all possible protospacers and target sites has been created and made available to the public (http://biodb.sdau.edu.cn/gc/index.html).

New plant breeding techniques have already proved successful in the potential improvement of apple and citrus fruit quality (Jia and Nian, 2014; Nishitani et al., 2016), although the feasibility of the technology has been exploited as proof-of-concept by the knockout of the *PDS* gene, acting on carotenoid biosynthesis at vegetative and reproductive levels. In contrast, to date, only two advanced studies in tomato have been described: precise targeting of the *pectate lyase* (*PL*) gene, which resulted in delayed fruit softening without perturbing other ripening-related parameters (Uluisik et al., 2016); and editing the *SlAGAMOUS-LIKE*6 (*SlAGL6*), a MADS-box transcription factor which provides tolerance to heat stress conditions and results in parthenocarpic fruits (Klap et al., 2016).

Taking into consideration the potential of these technologies, a more precise metabolic refinery is expected to come by selecting specific targets for nutritional and anti-nutritional molecules. This would imply the loss (knock out) and/or gain of function (activation) of selected enzymatic activities, respectively. Overall, these technologies potentially represent a powerful, innovative opportunity to introduce fine modifications in specific target genes. However, although the effect of knocking out genes has already proved successful, more work is needed for other kinds of gene remodeling (e.g., activation, production of allelic variants, etc.). To this end, significant contributions are likely to be provided by combining the CRISPR systems with additional enzymatic activities acting on DNA, such as recombinases, transposases, and DNA histone methyltransferases/acetyltransferases. These additional editing capabilities could potentially enable a vaster array of gene changes that, in the case of the fruit quality trait, may lead to a revolution in efficiency and respond better to consumer interests.

## Conclusion and perspectives

Three elements required to identify the genetic basis responsible for suitable phenotypes, and to use them to improve fruit quality produced in fields, have experienced huge technological progresses in the recent years. The first one is the constitution of germplasm banks in order to conserve the existing genetic diversity, including both natural and artificialy-induced variability. The second one is the ability to identify suitable phenotypes, notably innovations from wild genotypes, and to decipher their genetic basis. Finally, the third element is represented by the capacity to introduce the genetic elements into agronomic germplasm, remarkably through NPBT or selection assisted by markers. Altogether, the important advances in plant biotechnologies described in this review could last for long time, further facilitating plant breeding.

Indeed, biotechnologies are often praised for assuring food security to a growing Human population, through their impact on crop yield, and *de facto*, hunger has diminished drastically. Nevertheless, malnutrition still remains a global health problem, which also concerns developed countries (e.g., obesity) (FAO, 2015 hunger report; Steiber et al., 2004), suggesting that access to balanced and quality food is a combination of multiple factors besides agronomic yield as food allocation, waste and nutritional quality (Foley et al., 2011; Tilman and Clark, 2015). Hence, the responsibility of plant scientist is to develop solution in order to try to solve the society concerns. This could be achieved by a wide range of biotechnologies, dedicated to setting up the best suited genotypes, and producing knowledge that enables the optimization of agronomic practices (Chappell and LaValle, 2011; Amini et al., 2014).

However, in the context of recent societal mistrust about biotechnologies, sustainability of fruit production is becoming a quality trait more and more demanded by consumers, and awareness by research institutes. If one wants biotechnologies to be synonym of sustainability, improving yields and fruit quality in a long run on diverse field conditions, the notion of cost-benefits should be weighted ensuring that (i) Human and environmental health are not threatened, (ii) scientist and farmer self-reliance is not jeopardized by monopoles hold by international conglomerates including seed, chemical, and processing companies (Francis et al., 2003; Altieri and Nicholls, 2005; Chappell and LaValle, 2011; Guillemaud et al., 2016), and (iii) biotechnologies bring real benefits compared to existing processes (Temple et al., 2011; Abbo et al., 2014; Amini et al., 2014; Andersen et al., 2015; Reganold and Wachter, 2016). This debate around biotechnology use is well-illustrated by the debate around GMOs whose use could be more problematic than genetic manipulation itself (Altieri and Rosset, 1999; Chappell and LaValle, 2011; Amini et al., 2014; Guillemaud et al., 2016).

Therein, biotechnologies have their place within agroecology which bases the design of agricultural systems on the valorization of ecosystemic services to set up agri-food system economically viable, socially fair, and sustainable for the environment (Francis et al., 2003; Altieri and Nicholls, 2005; Wezel et al., 2009, 2014; García et al., 2013; Kershen, 2013). In this frame, evaluation of of biotechnologies relevance taking into account their global impact on all components of our societies, could be considered as a sustainable way to integrate biotechnologies to agriculture.

## Author contributions

QG, AF, and AG designed the perspective and all the authors wrote the manuscript.

## Funding

AF was provided by the Portuguese Foundation for Science and Technology (SFRH/BPD/100928/2014, FCT Investigator IF/00169/2015, PEst-OE/BIA/UI4046/2014), and to AG by the EC H2020 program (TRADITOM project 634561). QG benefited of the support of the Sunrise project ANR-11-BTBR-0005 funded by the ANR.

### Conflict of interest statement

The authors declare that the research was conducted in the absence of any commercial or financial relationships that could be construed as a potential conflict of interest.
